# Characterization of the Tubovesicular Network in *Plasmodium vivax* Liver Stage Hypnozoites and Schizonts

**DOI:** 10.3389/fcimb.2021.687019

**Published:** 2021-06-14

**Authors:** Kayla Sylvester, Steven P. Maher, Dora Posfai, Michael K. Tran, McKenna C. Crawford, Amélie Vantaux, Benoît Witkowski, Dennis E. Kyle, Emily R. Derbyshire

**Affiliations:** ^1^ Department of Molecular Genetics and Microbiology, Duke University Medical Center, Durham, NC, United States; ^2^ Center for Tropical and Emerging Global Diseases, University of Georgia, Athens, GA, United States; ^3^ Chemistry Department, Duke University, Durham, NC, United States; ^4^ Malaria Molecular Epidemiology Unit, Pasteur Institute in Cambodia, Phnom Penh, Cambodia

**Keywords:** *Plasmodium*, malaria, *vivax*, hypnozoites, tubovesicular network, AQP3

## Abstract

*Plasmodium* is a genus of apicomplexan parasites which replicate in the liver before causing malaria. *Plasmodium vivax* can also persist in the liver as dormant hypnozoites and cause clinical relapse upon activation, but the molecular mechanisms leading to activation have yet to be discovered. In this study, we use high-resolution microscopy to characterize temporal changes of the *P. vivax* liver stage tubovesicular network (TVN), a parasitophorous vacuole membrane (PVM)-derived network within the host cytosol. We observe extended membrane clusters, tubules, and TVN-derived vesicles present throughout *P. vivax* liver stage development. Additionally, we demonstrate an unexpected presence of the TVN in hypnozoites and observe some association of this network to host nuclei. We also reveal that the host water and solute channel aquaporin-3 (AQP3) associates with TVN-derived vesicles and extended membrane clusters. AQP3 has been previously shown to localize to the PVM of *P. vivax* hypnozoites and liver schizonts but has not yet been shown in association to the TVN. Our results highlight host-parasite interactions occur in both dormant and replicating liver stage *P. vivax* forms and implicate AQP3 function during this time. Together, these findings enhance our understanding of *P. vivax* liver stage biology through characterization of the TVN with an emphasis on the presence of this network in dormant hypnozoites.

## Introduction

Apicomplexans constitute a large group of parasitic protozoans that cause diseases, including the five species of *Plasmodium* that cause human malaria. Human infection by *Plasmodium* begins when a female *Anopheles* mosquito deposits sporozoites into the bloodstream which then migrate to the liver. Once in a hepatocyte, sporozoites transform and rapidly replicate to yield tens of thousands of merozoites from a single schizont (Frischknecht et al., 2004; Prudêncio et al., 2006; Ejigiri and Sinnis, 2009; Ploemen et al., 2009). This stage is asymptomatic but a prerequisite to the infection of red blood cells (RBCs) that leads to disease. Many species of *Plasmodium*, including *P. falciparum* and *P. berghei*, follow this path, while others such as *P. vivax* can differentiate in hepatocytes into either schizonts or hypnozoites, a biologically quiescent form (Krotoski et al., 1982). Dormant hypnozoites cause recurrent blood stage infections (relapses) upon activation, effectively sustaining repeated blood infection and furthering transmission (Krotoski et al., 1982; Mikolajczak et al., 2015). *P. vivax* hypnozoites are insensitive to most antimalarials except 8-aminoquinolines, which are contraindicated in populations with glucose-6-phosphate dehydrogenase (G6PD) deficiency, highlighting the need for new anti-hypnozoite agents (Howes et al., 2012; Lu and Derbyshire, 2020). Unfortunately, efforts to identify compounds capable of inhibiting hypnozoites are hampered by our limited understanding of the molecular pathways that enable parasite survival and activation during this stage.

To successfully develop and replicate within their host, *Plasmodium* resides within a membrane-bound compartment formed during invasion termed the parasitophorous vacuolar membrane (PVM) (Meis et al., 1983). Derived from the host membrane itself, the PVM serves as the host-pathogen interface and dynamically changes to facilitate development by enabling nutrient acquisition and protecting the parasite from apoptosis (Van De Sand et al., 2005; Van Dijk et al., 2005; Kaushansky et al., 2013; Meireles et al., 2017; Sá E Cunha et al., 2017). For example, the PVM diameter can expand beyond that of the pre-invasion hepatocyte membrane diameter and can recruit both host and parasite proteins. The precise composition of this membrane remains unknown, but several studies have revealed it changes minutes to hours after liver invasion when parasite proteins, including up-regulated in infective sporozoites gene 4 (UIS4), are translocated to the PVM (Mueller et al., 2005; Kaushansky and Kappe, 2015; Prado et al., 2015; Sá E Cunha et al., 2017; Schnider et al., 2018). While fewer host proteins are known to associate with the PVM after invasion, examples of protein recruitment crucial for host defense and parasite development have been reported (Grützke et al., 2014; Prado et al., 2015; Thieleke-Matos et al., 2016; Wacker et al., 2017; Posfai et al., 2018; LaMonte et al., 2019; Niklaus et al., 2019; Raphemot et al., 2019; Posfai et al., 2020).

A key component of the host-parasite interactions in the *Plasmodium* liver stage is the physical alteration of the PVM. Several elegant studies have shown that the PVM can extend into the host hepatocyte with a highly dynamic membranous system, the tubovesicular network (TVN) (Grützke et al., 2014; Agop-Nersesian et al., 2017; Agop-Nersesian et al., 2018; Niklaus et al., 2019). In the *P. berghei* liver stage, this system consists of extended membrane clusters, tubules, and vesicles that move to and from the PVM (Grützke et al., 2014). The TVN expansion into the host cytosol is proposed to aid in nutrient acquisition and immune evasion, imparting key survival strategies to the parasite (Grützke et al., 2014; Agop-Nersesian et al., 2017; Agop-Nersesian et al., 2018; Niklaus et al., 2019). While UIS4 is an established parasite-derived *P. berghei* TVN marker, the only host proteins reported to associate with TVN features are LC3, LAMP1, p62 and CD63 (Grützke et al., 2014; Agop-Nersesian et al., 2017; Agop-Nersesian et al., 2018; Niklaus et al., 2019), which have been exclusively studied in mouse-infective *Plasmodium*. There has been no evidence of a host protein responsible for nutrient uptake to be associated with the TVN. In the blood stage, the TVN is complemented by an unusual protein trafficking system that involves long-known parasite-derived membranous structures in the RBC cytosol, termed Maurer’s clefts in *P. falciparum* and Schüffner’s dots in *P. vivax* (Schüffner, 1899; Alkawa et al., 1975; Wickert et al., 2004; Spycher et al., 2006; Wickert and Krohne, 2007; Tamez et al., 2008; Akinyi et al., 2012; Mundwiler-Pachlatko and Beck, 2013; Tokumasu et al., 2014; Sakaguchi et al., 2016). Notably, these structures have not been found in infected hepatocytes. Additionally, the TVN has not yet been reported in liver stage *P. vivax*, likely due to the technical difficulties associated with studying parasites during this stage.

Recent advances in *P. vivax* model systems that can support liver stage schizont and hypnozoite development have been instrumental to setting the stage for anti-hypnozoite drug screens and aiding molecular studies to understand parasite biology (March et al., 2013; Mikolajczak et al., 2015; Antonova-Koch et al., 2018; Gural et al., 2018; Roth et al., 2018; Maher et al., 2020). Here, we analyzed *P. vivax*-infected primary human hepatocytes (PHH) to discover and characterize TVN features throughout infection (Roth et al., 2018). Interestingly, we observe that TVN+ exoerythrocytic forms (EEFs) are primarily dormant hypnozoites as opposed to replicating schizonts at 8 days post infection (dpi). We also found that the host water and solute channel aquaporin 3 (AQP3) co-localizes with TVN-derived vesicles and extended membrane clusters at certain time points in *P. vivax* hypnozoites as well as liver stage *P. vivax* and *P. berghei* schizonts. AQP3 recruitment to the *Plasmodium* PVM has been previously reported (Posfai et al., 2018; Posfai et al., 2020), but the current discovery of TVN association suggests an as yet unknown function in this important network that enables interactions between the host and pathogen. Together, our longitudinal analysis characterizes the TVN in *P. vivax* liver stage for the first time and identifies the host protein AQP3 as associated with this network.

## Materials and Methods

### Lead Contact and Materials Availability

Further information and requests for *P. vivax-*related reagents should be directed to Benoît Witkowski (bwitkowski@pasteur-kh.org). There are restrictions on the availability of some *P.* vivax-related reagents due to inadequate methodology for the preservation and propagation of the parasites in clinical isolates. This study did not generate new unique reagents from Emily Derbyshire. Further information about auphen and imaging reagents should be directed to and will be fulfilled by the Lead Contact, Emily Derbyshire (emily.derbyshire@duke.edu).

### *P. vivax* Infections of Primary Human Hepatocytes (PHH)

*P. vivax* infections were completed as previously described (Roth et al., 2018). Blood samples were collected from symptomatic *P. vivax* patients at local health facilities in Mondulkiri province (eastern Cambodia) from 2018-2019. Patients presenting signs of severe malaria, infected with non-vivax malaria parasites, under 5 years of age, or who were pregnant or lactating were excluded from the collection. Following informed consent from eligible study participants, venous blood samples were collected by venipuncture into heparin-containing tubes (Beckton Dickinson, Cat# 367886). Venous blood was pelleted and serum was replaced with naïve human serum (Interstate Blood bank, Inc.) before feeding to *An. dirus* mosquitoes using glass bell insect feeders held at 37°C. Following a *P. vivax* gametocyte-containing bloodmeal, *An. dirus* mosquitoes were maintained on a natural light cycle and 10% sucrose in water. Mosquitoes found positive for *P. vivax* oocysts at six days post-feeding were transported to the IPC facility in Phnom Penh, Cambodia where salivary glands were aseptically dissected into RPMI without sodium bicarbonate (Gibco, Cat# 61870-010) on day 16-21 post-feeding. Cryopreserved PHH were thawed into InVitroGro™ CP Medium (BioIVT) including a 1x antibiotic mixture (PSN, Gibco and Gentamicin, Gibco) and 18,000 live cells were added to selected wells of a collagen-coated 384-well plate (Grenier). Cultures were maintained in a standard tissue culture incubator at 37°C and 5% CO_2_. Two lots of PHH were used for IFA analysis: lot BGW was obtained from a 50-year-old Caucasian male, lot UBV was obtained from a 57-year old Caucasian male. Infection of PHH was performed at 2 days post-seed by diluting freshly dissected sporozoites into CP media with antibiotics, adding 20 μL sporozoite-media mixture to each well, and centrifugation the 384-well plate at 200 RCF for 5 min at room temperature. Media was exchanged with fresh CP media containing antibiotics the day after infection and every 2-3 days thereafter.

### Liver Stage *P. vivax* Immunofluorescent (IFA) Microscopy and TVN Analysis

*P. vivax*-infected PHH were fixed with 4% paraformaldehyde (ThermoFisher) for 1 hr. Fixed cells were stained with recombinant mouse anti-*P. vivax* Upregulated in Infectious Sporozoites-4 antibody (rPvUIS4, (Schafer et al., 2018)) (1:2,500) in staining buffer (0.03% TritonX-100, 1% (w/v) BSA in PBS) overnight at 4°C. Cells were then washed with PBS and stained with goat anti-mouse Alexa Fluor^®^ 488-conjugated antibody (1:1000) in staining buffer overnight at 4°C. Cells were then washed with PBS, stained with rabbit anti-*Hs*AQP3 (AbClonal, Rockland) (1:200) for 48 hrs at 4°C, washed again with PBS and incubated with donkey anti-rabbit Alexa Fluor^®^ 568-conjugated antibody (ThermoFisher) (1:400). Stained cells were washed with PBS and counterstained with 0.5 μg/mL DAPI (ThermoFisher). Control wells were stained as above with omission of rabbit anti-*Hs*AQP3 (AbClonal) staining (**Supplemental Figure 5A**). Fluorescence was detected using a Zeiss 880 Airyscan inverted confocal and images were analyzed using ImageJ (Schindelin et al., 2012). Infections were evaluated as 2D and Z-stacked images to assess TVN structures present. Vesicles are defined as UIS4+ staining that are detached from PVM staining, while tubules are classified as a thin feature protruding into the host cytosol with a length greater than 1 µm and width less than 0.5 µm at the PVM interface. Extended membrane clusters are characterized by non-prominence staining that extends to the host cytosol from the PVM, which are elongated and coarser than then PVM. To ensure a non-bias approach, 3–4 researchers individually scored each EEF for features present. Individual scores were compared, and the few discrepancies noted were discussed to achieve a consensus before finalizing. AQP3 colocalization with TVN structures was observed throughout *P. vivax* liver stage infection (2–10 dpi). GraphPad Prism was used to generate quantitative figures.

### *P. berghei* Infections of HuH7 Cells

HuH7 cells (kind gift from Dr. Peter Sorger) were cultured in DMEM + L-Glutamine (Gibco) supplemented with 10% (v/v) heat-inactivated FBS (HIFBS) (Sigma) and 1% (v/v) antibiotic/antimycotic (Sigma). Cultures were maintained in a standard tissue culture incubator at 37°C and 5% CO_2_. HuH7 ΔAQP3 mut1 cells have a 39 bp deletion in exon 2 of AQP3 resulting loss of functional protein (Posfai et al., 2018). These cells were utilized in comparison to HuH7 wild type cells and maintained as described above. *Anopheles stephensi* mosquitoes infected with luciferase-expressing *P. berghei* ANKA were purchased from NYU Langone Medical Center Insectary. GFP-expression *P. berghei* ANKA from NYU were utilized for infection with HuH7 and HuH7 ΔAQP3 cells. Cells were seeded (2.5x10^5^/well) into a 24-well plate with coverslips 24 hours prior to infection. Cells were infected with ~50,000 *P. berghei* sporozoites per well that were freshly dissected from *An. stephensi* mosquitoes.

### Liver Stage *P. berghei* IFA Microscopy and TVN Analysis

*P. berghei*-infected HuH7 cells and *P. berghei*-infected HuH7 ΔAQP3 were fixed at 48 hpi with 3% paraformaldehyde at room temperature for 15 min. Cells were permeabilized with 0.2% TritonX for 10 min, washed with PBS and blocked with 3% BSA for 1 hr at room temperature. Cells were stained with goat anti-*Pb*UIS4 (antibodies-online) (1:1000) for 1 hr at room temperature, washed with PBS and incubated with secondary donkey anti-goat Alexa Fluro^®^ 488-conjugated antibody (ThermoFisher) (1:400). Cells were then stained sequentially with rabbit anti-AQP3 (Rockland) (1:100) overnight at 4°C and donkey anti-rabbit (ThermoFisher) (1:400) for 1 hr at room temperature. Lastly, cells were stained with DAPI. Control wells were stained as above with omission of rabbit anti-*Hs*AQP3 staining (**Supplemental Figure 5D**) or omission of secondary donkey anti-goat Alexa Fluro^®^ 488-conjugated antibody (ThermoFisher) (**Supplemental Figure 5E**). Fluorescence was detected using a Zeiss Axio Observer Widefield Fluorescence Microscope or Zeiss 880 Airyscan inverted confocal, and images were analyzed using ImageJ.

## Results

### *P. vivax* TVN Characterization

**Figure 1:**
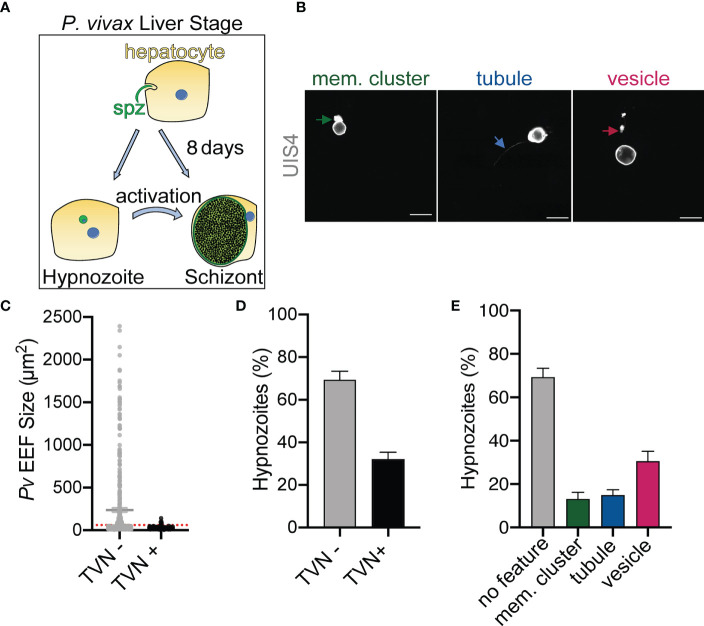
*P. vivax*-infected PHH contain TVN features. **(A)** Schematic representing *P. vivax* liver stage infection. **(B)** Examples of TVN features including extended membrane cluster (mem. cluster, green arrow), tubule (blue arrow) and vesicles (magenta arrow) in *P. vivax*-infected PHH. Cells stained with anti-*Pv*UIS4 (grey). Scale bar is 10 µm. **(C)** Relative size of *P. vivax* EEFs that are TVN- (grey) or TVN+ (black). Red dotted line indicates cut off for hypnozoites (60 µm^2^). **(D)** Percentage of TVN- (grey bar) or TVN+ (black bar) *P. vivax* hypnozoites. **(E)** Characterization of *P. vivax* hypnozoites containing TVN+ or TVN- EEFs. Percentage of each characteristic including extended membrane cluster (mem. cluster, green bar), tubule (blue bar), and vesicle (magenta bar) is indicated. Grey bar indicates percentage of TVN- hypnozoites. **(B–E)** Assessed at 8 dpi. Data reported as mean ± SEM (3 independent wells assessed with >200 EEFs, n=2 biological replicates).

**Figure 2:**
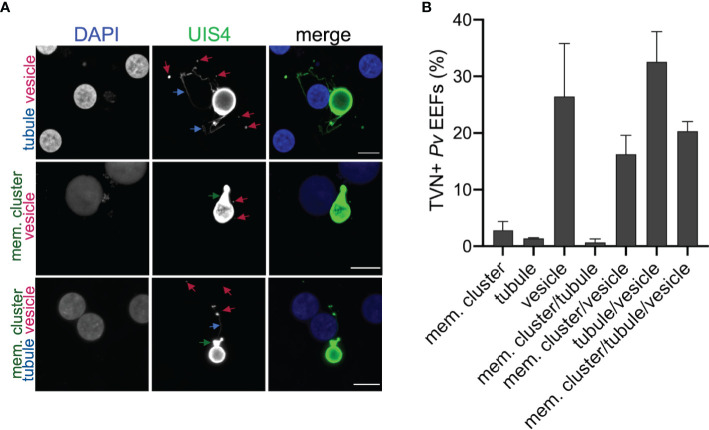
*P. vivax* TVN phenotypes. **(A)** Representative confocal images of *P. vivax* EEFs with TVN features in PHH at 8 dpi. Cells stained with DAPI (blue) and anti-*Pv*UIS4 (green). TVN features observed indicated as extended membrane cluster (mem. cluster, green arrow), tubule (blue arrow), and vesicle (magenta arrow). Scale bar is 10 µm. **(B)** Characterization of EEFs displaying one or more TVN features on 8 dpi. Data reported as mean ± SEM (3 independent wells assessed with >200 parasites, n=2 biological replicates).

The *P. berghei* liver stage TVN is known to be important for nutrient acquisition and immune evasion, but this network has not yet been characterized in the *P. vivax* liver stages. *P. berghei* requires approximately 2 days to transition from a sporozoite to a mature schizont in the liver, while *P. vivax* schizont maturation occurs over 8-10 days *in vitro*. *P. vivax* also has an alternative morphological and physiological route that arrests development to create a quiescent hypnozoite that can activate days or months after infection (**Figure 1A**). To examine the possibility of TVN formation in *P. vivax* schizonts or hypnozoites, we used high-resolution confocal microscopy to obtain images of *P. vivax*-infected PHH 8 dpi. Parasites were stained with a recombinant anti-*Plasmodium* UIS4 (rUIS4) antibody (Schafer et al., 2018) to visualize the PVM/TVN and DAPI to evaluate number of parasite nuclei. The TVN+ EEFs demonstrated through this imaging at 8 dpi included features classified as extended membrane clusters, tubules and TVN-derived vesicles (**Figure 1B**) similar to a previous liver stage TVN *P. berghei* study (Grützke et al., 2014). Any EEF demonstrating any of these features was classified as TVN+, while EEFs containing no UIS4+ TVN structures were classified as TVN-. Schizont and hypnozoite populations are of similar sizes and are difficult to distinguish before 6 dpi, but our analysis suggested TVN features were more abundant in smaller sized parasites when observed at 8 dpi. To explore this observation, we performed two classifications. First, we measured the area of each EEF and divided them into one of two categories: TVN- or TVN+ EEFs at 8 dpi (**Figure 1C**). This analysis revealed that the vast majority of TVN+ *P. vivax* EEFs were relatively small at 8 dpi. Second, to definitively characterize EEF populations as hypnozoites and schizonts, we quantified the number of parasite nuclei and growth area of each EEF when imaged at high resolution (63x magnification). A uninucleated parasite indicates a non-replicating dormant hypnozoite, while a multinucleated parasite indicates a schizont or activated hypnozoite, as either would be actively replicating (Mikolajczak et al., 2015). At this magnification we observed all multinucleated EEFs as having an area ≥75 μm^2^, thus we established a strict size cutoff for hypnozoites as having a growth area <60 µm^2^ (**Supplemental Figure 1A**) and for schizonts as having a growth area above >200 µm^2^ at 8 dpi (**Supplemental Figure 1B**). While few EEFs were noted with a growth area between 60 µm^2^ and 200 µm^2^ at this time, these forms were excluded from classification of hypnozoite or schizont to ensure no ambiguity between the two forms. This more precise analysis of the small parasite population revealed the vast majority of TVN+ EEFs were classified as hypnozoites. Using these metrics, we found that 32% of hypnozoites had one or more TVN features at 8 dpi (**Figure 1D**), while all schizonts were TVN- among the 529 EEFs analyzed on this dpi. We further examined TVN+ EEFs at multiple focal planes (z-stacks) to ensure detection of all features. This analysis revealed that TVN-derived vesicles in the host cytosol were most prominent in cells containing hypnozoites at 8 dpi, present within 30% of the population (**Figure 1E**). Extended membrane clusters and tubules were present in ~15% of the hypnozoite population. Interestingly, through our analysis we observed that a majority of *P. vivax* liver stage TVN+ EEFs had multiple features (**Figure 2** and **Supplemental Figure 2**). It is also notable that most TVN+ EEFs contained vesicles (**Figure 2B**).

**Figure 3:**
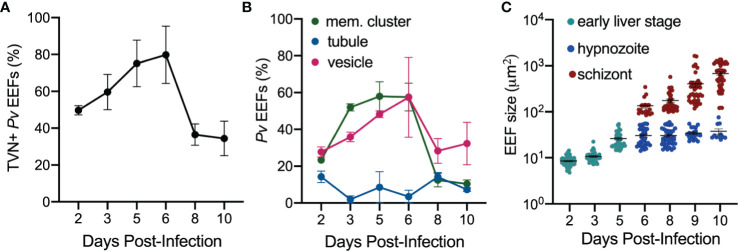
Characterization of *P. vivax* TVN throughout infection. **(A)** Percentage of *P. vivax* TVN+ EEFs (black) throughout infection of PHH (2–10 dpi). **(B)** Percentage of total *P. vivax* EEFs with indicated TVN characteristic days 2–10 post-infection of PHH. TVN+ EEFs are classified into exhibiting extended membrane clusters (mem. clusters, green), tubules (blue), and TVN-derived vesicles (vesicles, magenta). **(C)** *P. vivax* EEF size days 2–10 post-infection of PHH. Differentiation is shown between early liver stage (teal), hypnozoites (blue), and schizonts (red). Data reported as mean area ± SEM for each stage (early liver stage, hypnozoite, and schizont) per day. **(A, B)** Data reported as mean ± SEM (>30 EEFs each day, n=2 biological replicates).

**Figure 4:**
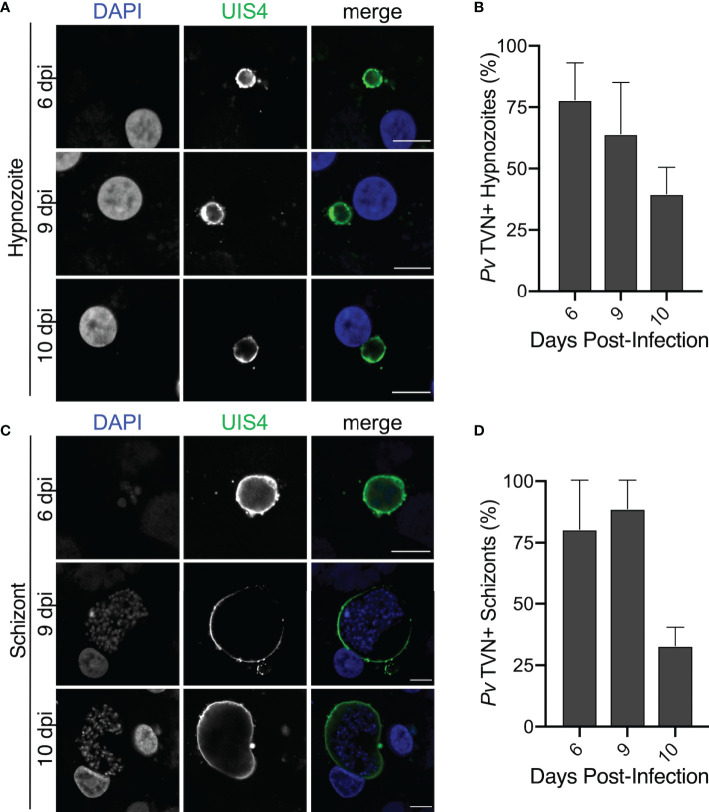
TVN features in *P. vivax* hypnozoites and schizonts. **(A)** Representative confocal images of *P. vivax *TVN+ hypnozoites in PHH at 6, 9, and 10 dpi. Host and parasite nuclei stained with DAPI (blue) and parasite PVM stained with anti-*Pv*UIS4 (green). Scale bar is 10 µm. **(B)** Percent of TVN+ hypnozoites at 6, 9, and 10 dpi. Data reported as mean ± SEM. **(C)** Confocal images of TVN+ schizonts in PHH at 6, 9, and 10 dpi. Host and parasite nuclei stain with DAPI (blue) and *Plasmodium* PVM stained with anti-*Pv*UIS4 (green). Scale bar is 10 µm. **(D)** Percent of TVN+ schizonts at 6, 9, and 10 dpi. Data reported as mean ± SEM. **(A–D)** >50 EEFs each day, n=2 biological replicates.

We hypothesized that TVN features may vary depending on the parasite’s developmental stage. Therefore, we took a longitudinal approach to our analysis and evaluated TVN features every day of a 10-day infection of PHH with *P. vivax*. This analysis showed that TVN+ parasites were present throughout PHH infection with *P. vivax*, with a higher percentage of TVN+ EEFs observed on days 5 and 6 post-infection (**Figure 3A**). In *P. berghei*, TVN+ EEFs are more prevalent at early times of hepatocyte infection, appearing within 30 min and being most abundant between 6–24 hours post-infection (hpi) (Grützke et al., 2014). Here, we observe TVN features at early *P. vivax* EEF stages (2–3 dpi), where extended membrane clusters and TVN-derived vesicles increase in abundance from 2 to 6 dpi (**Figure 3B**). Based on this analysis, 3–5 and 6–8 dpi are particularly dynamic times where the greatest changes in TVN abundance and composition were observed. For example, a remarkable decrease in extended membrane clusters is observed between 6 to 8 dpi, such that less are present at 8 dpi than at the early time points (i.e., 2 dpi). While a decrease in TVN-derived vesicles was also observed between 6 to 8 dpi, the prevalence of this feature within cells remained high compared to that of extended membrane clusters. TVN-derived vesicles were maintained in >20% of EEFs throughout the course of infection. These TVN-derived vesicles are sometimes seen close to the EEF but can also be found at distal parts of the cell (**Figure 2**, **Supplemental Figure 2**). Relatively less change in tubule abundance was observed throughout intrahepatic *P. vivax* development. These tubules varied in length and interestingly, we observed some instances of tubule association with the host nuclei (**Supplemental Figure 3**, **Video S1**). Generally, the abundance of EEFs with one or multiple TVN features varied throughout the 10-day infection (**Supplemental Figure 4**). Hypnozoites and schizonts are distinguishable by 3 dpi *in vivo* (Mikolajczak et al., 2015), but we found we could not confidently distinguish between these forms until day 6 post infection. On day 5, a single EEF population was observed based on size (area < 65 μm^2^) and presence of 1 parasite nucleus. However, on day 6 we observed two EEF populations; multinucleated EEFs at >75 μm^2^ and uninucleated EEFs at < 60 μm^2^ (**Figure 3C**). On days 6–10 post-infection, when schizonts can be distinguished from hypnozoites (**Figure 3C**), we evaluated TVN features as a function of EEF size. Though we did not observe TVN features in schizonts at 8 dpi, we observed a TVN-positive population of schizonts and hypnozoites at 6, 9 and 10 dpi. Specifically, the percentage of TVN+ EEFs in both hypnozoites and schizonts was greater at 6 dpi when compared to 10 dpi (**Figure 4**).

### AQP3 Is Associated With the *Plasmodium* TVN

**Figure 5:**
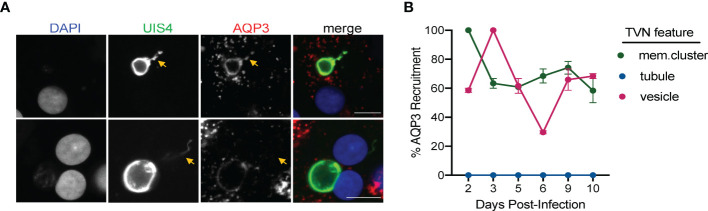
Localization and recruitment of AQP3 to TVN features during *P. vivax* infection. **(A)** Representative confocal images of *P. vivax* EEFs exhibiting TVN features, stained with DAPI (blue), anti-*Pv*UIS4 (green), and anti-*Hs*AQP3 (red) at 8 dpi. *P. vivax*-infected PHH show localization (top) and no localization (bottom) of AQP3 to specific TVN features (yellow arrows). Scale bar is 10 µm. **(B)** Relative percentage of TVN features in *P. vivax*-infected PHH exhibiting AQP3 recruitment. Data reported as mean ± SEM (2–10 dpi, >30 EEFs per dpi, n=2 biological replicates).

The precise compositions of the *Plasmodium* liver stage PVM and TVN remain to be resolved. We recently established that host AQP3 is recruited to the PVM of *P. berghei* (Posfai et al., 2018) and *P. vivax* EEFs, including both developing schizonts and dormant hypnozoites (Posfai et al., 2018; Posfai et al., 2020). To better understand AQP3 recruitment to these different forms, we visualized *P. berghei*-infected HuH7 cells and *P. vivax*-infected PHH after staining with DAPI, anti-rUIS4 and anti-human AQP3 antibodies. In addition to evaluating AQP3 staining with primary and secondary antibody controls, the AQP3 antibody was validated using HuH7 cells with CRISPR disruption of AQP3 (**Supplemental Figure 5**) (Posfai et al., 2018). No cross reactivity was observed between primary and secondary antibodies used in this study and the antibody specificity is supported by the lack of AQP3 staining to *P. berghei* EEFs in ΔAQP3 cells compared to wild-type cells. After analyzing our immunofluorescence microscopy images, we observed that AQP3 co-localizes to some, but not all TVN features in *P. berghei* and *P. vivax* EEFs at 2 and 8 dpi, respectively (**Figure 5A** and **Supplemental Figure 6**). Similar to a previous report, we also observed AQP3 co-localization to the PVM (Posfai et al., 2018; Posfai et al., 2020). To further investigate AQP3 recruitment to the TVN, we analyzed *P. vivax* EEFs days 2–10 post-infection (**Figure 5B**). At early time points (2–3 dpi), AQP3 is not observed in every *P. vivax* EEF (Posfai et al., 2020); however, it was highly recruited to extended membrane clusters and vesicles when present. For example, when an EEF exhibited both AQP3 and extended membrane TVN features at 2 dpi, they were observed together in every EEF examined in our analysis. Likewise, AQP3 was highly correlated with TVN-derived vesicles at 3 dpi. Notably, we observed no AQP3 recruitment to TVN features in EEFs that lacked protein recruitment to the PVM. Further, we found no instance of AQP3 co-localizing with *P. vivax* TVN tubules at any day post infection despite looking in multiple planes (z-stacks).

## Discussion

*Plasmodium vivax* remains a critical hurdle to malaria eradication efforts due in large part to the parasite’s ability to persist in the liver and lead to relapses in blood infections. During the liver stage, the parasites can mature into schizonts after invasion or delay replication as a seemingly arrested form termed a hypnozoite, which can unpredictively activate. Processes that drive invasion, maturation and development during the liver stage are considered elusive, yet much less is understood about contributing factors that enable the survival of dormant hypnozoites. Our study provides the first characterization of the TVN in *P. vivax* and suggests a role for this network in liver stage hypnozoites and schizonts. Previously well-characterized in liver stage *P. berghei* EEFs, the TVN is proposed to be critical for nutrient acquisition and evasion of the host immune responses (Mueller et al., 2005; Agop-Nersesian et al., 2017; Agop-Nersesian et al., 2018; Niklaus et al., 2019).

The TVN in developing *P. vivax* EEFs was anticipated based on *P. berghei* studies, but its presence in hypnozoites, which are considered biologically quiescent, was less expected. In fact, at 8 dpi TVN+ EEFs were more prevalent in *P. vivax* hypnozoites versus schizonts. This presence of the TVN in hypnozoites seems counterintuitive to notions of the dormant state and suggests that many as yet unknown complex host-parasite interactions occur during this time. Indeed, recent RNA-sequencing studies of *P. vivax* hypnozoites have suggested metabolic activity occurs at this time (Gural et al., 2018). We observe TVN features present in some hypnozoites but not all, suggesting different hypnozoite populations. One explanation for these TVN features is that they are present in hypnozoites that are primed for activation, potentially for influx of nutrients before nuclear division. Based on previous research, hypnozoites are capable of activating *in vitro* after 1–2 weeks (Voorberg-van der Wel et al., 2013; Gupta et al., 2019). Additionally, we observe a population of TVN+ schizonts that are between 75–200 μm^2^ in EEF size at 9 and 10 dpi, suggesting newly replicating (i.e., activated) parasites. It is possible that in this *in vitro* system these schizonts arose from a sub-population of TVN+ hypnozoites that is not fully dormant and therefore is ‘activated’ more quickly. Future studies utilizing an anti-LISP2 antibody would facilitate differentiation between these populations as it discerns hypnozoites (LISP-) and newly developing EEFs (LISP+) (Gupta et al., 2019). Alternatively, it is possible that the EEFs, including hypnozoites, displaying TVN features are able to better evade the host immune response. This proposed function could support the long latency of some *P. vivax* hypnozoites and their ability to avoid clearance from the host cell. These hypotheses could be further explored by evaluating host and parasite gene expression in TVN+ and TVN- EEF populations as well as the association of host immune response proteins to the TVN.

Interestingly, we observed differences in the utilization of this network throughout infection between *P. vivax* and *P. berghei.* Both *Plasmodium* species exhibit a similar sequence of developmental events that lead to schizonts, which includes invasion, morphological changes, replication, merozoite formation, and parasite release, though at varying time scales. Here, we observe a moderately different proportion of *P. vivax* TVN+ EEFs (35–80%) compared to previous findings with *P. berghei* (~60–90%). This variation could be due to the time points analyzed or differences in studying fixed versus live cells. Of note, our study would not detect UIS4- TVN features, since we utilize this protein as a PVM/TVN marker based on previous *P. berghei* studies, and it is not yet established if UIS4 is present in all *P. vivax* TVN features. In *P. berghei*, TVN activity increases before replication (Grützke et al., 2014), at early time points, and we similarly observe a rise in TVN+ EEFs before nuclear division between 2–6 dpi. Interestingly, we observe TVN+ schizonts, with TVN-derived vesicles being the most prominent feature, at days 9 and 10, but none on day 8. At these later stages, the TVN may facilitate egress or further protect the parasite from clearance by shedding autophagy related proteins to these TVN-derived vesicles, as is the case with *P. berghei* (Agop-Nersesian et al., 2017; Agop-Nersesian et al., 2018; Niklaus et al., 2019). A comprehensive time course that monitors activation, egress and TVN feature dynamics within a single live cell would enhance our understanding of TVN function in *P. vivax*. In addition to correlating the TVN with developmental changes and/or host clearance, this study would reveal the role of the PVM prominence, characterized by crescent shaped UIS4 staining (Mikolajczak et al., 2015), in this network. We aimed to examine the possible correlation of the prominence with the TVN but did not observe a clear association. Though the proposed real-time experiments would provide valuable insights, they remain difficult as the genetic tools necessary to generate transgenic *P. vivax* sporozoites that express fluorescently tagged UIS4 are not yet available.

We observed host AQP3, a protein channel that is permeable to water, glycerol and other small solutes (Echevarria et al., 1994; Ishibashi et al., 1994; Echevarría et al., 1996; Meinild et al., 1998; Beitz et al., 2006; Hara-Chikuma et al., 2015; Thiagarajah et al., 2017), associates with the TVN in both *P. berghei* and *P. vivax* EEFs. We have previously demonstrated *AQP3* upregulation after *P. berghei* infection of liver cells and reduced intrahepatic *P. berghei* size after genetic disruption of the protein (Posfai et al., 2018). A well-characterized role for AQP3 in mammalian cells is in nutrient flux, a function similar to that of the TVN in liver stage *P. berghei* EEFs (Agop-Nersesian et al., 2018). We detected AQP3 exclusively in extended membrane clusters and TVN-derived vesicles in *P. vivax* EEFs throughout infection, suggesting important and coincident functions. Notably, we did not observe localization of AQP3 to all extended membrane clusters and vesicles. It remains unknown if AQP3 is trafficked from the host cytosol to the PVM by TVN-derived vesicles or alternatively, AQP3 in the PVM is shed into vesicles. In support of the former hypothesis, we did not observe AQP3 localization in TVN-derived vesicles without localization to PVM concurrently. Among the host proteins known to associate with the liver stage *P. berghei* TVN, it has been reported that both LC3 and LAMP1 are shed from the PVM to the TVN to facilitate evasion of the host immune response (Agop-Nersesian et al., 2017; Niklaus et al., 2019). While this suggests a role for the TVN in protection from the host response, the network is also known to be important for nutrient acquisition. Previous reports have shown that host-derived endosomes and lysosomes associate with the TVN, presumably releasing their degraded contents to the network where they can then potentially be imported as nutrients (Grützke et al., 2014; Agop-Nersesian et al., 2017). AQP3 could therefore facilitate this nutrient acquisition. The *Plasmodium* nutrient channel exported protein 2 (EXP2) (Garten et al., 2018) has also been observed in the PVM/TVN of liver stage *P.vivax* schizonts (Roth et al., 2018; Posfai et al., 2020), and other *Plasmodium* spp. (Matz et al., 2015; Kalanon et al., 2016), but is absent from *P. vivax* hypnozoites (Roth et al., 2018). Thus, AQP3 may support nutrient acquisition in TVN+ hypnozoites to enable growth (without replication), as EXP2 is not present to fulfill this role. But future genetic and chemical tools are necessary to establish the function of host AQP3 in the TVN and PVM of liver stage schizonts and hypnozoites.

Taken together, this study characterizes the TVN of *P. vivax* throughout liver stage infection and demonstrates that schizonts and hypnozoites contain TVN-derived vesicles, tubules and extended membrane clusters. In particular, we propose a previously unknown role for the TVN in dormant *P. vivax* hypnozoites and highlight the possible function of AQP3 in this network in both mouse- and human-infective liver stage *Plasmodium*. Future efforts to resolve the molecular function of the TVN in *P. vivax* throughout schizont and hypnozoite development as well as studies to map host and parasite constituents of the TVN will be critical to advancing our understanding of host-parasite interactions during this critical time of the life cycle.

## Data Availability Statement

The original contributions presented in the study are included in the article/**Supplementary Material**. Further inquiries can be directed to the corresponding author.

## Ethics Statement

Clinical isolate collection and research procedures were reviewed and approved by the Cambodian National Ethics Committee for Health Research (approval number: #101NECHR, #270NECHR & #273NECHR). Confirmed written consent from all human subjects was obtained by all volunteers and patients.

## Author Contributions

Conceptualization, KS, SM, DK, and ED. Methodology, KS, SM, DP, AV, KS, and BW. Formal Analysis, KS, MT, MC, and ED. Resources, SM, AV, BW, DK, and ED. Writing – Original Draft, KS and ED. Writing – Review and Editing, KS, SM, DP, MT, MC, AV, BW, and ED. Visualization, KS, and ED. Supervision, SM, AV, BW, DK, and ED. Funding Acquisition, SM, DK, and ED. All authors contributed to the article and approved the submitted version.

## Funding

Work was supported by the Bill & Melinda Gates Foundation (OPP1023643 to DK), the NIH (1DP2AI138239 to ED) for laboratory support, and fellowship support by the NSF (DGE-1644868, to KS).

## Conflict of Interest

The authors declare that the research was conducted in the absence of any commercial or financial relationships that could be construed as a potential conflict of interest.

## Supplementary Material

Supplementary Figure 1
*P. vivax* variation in size 8 dpi. **(A)** Representative confocal images of *P. vivax* hypnozoites in PHH at 8 dpi, defined by EEF area < 60 µm^2^. **(B)** Representative confocal images of *P. vivax* schizonts in PHH at 8 dpi, defined by EEF area > 200 µm^2^. **(A, B)** Cells stained with DAPI (blue) and anti-*Pv*UIS4 (green). Scale bar is 10 µm.

Supplementary Figure 2
*P. vivax* TVN+ EEFs contain multiple features. Confocal images of *P. vivax* EEFs with TVN features in PHH at 8 dpi. Cells stained with DAPI (blue) and anti-*Pv*UIS4 (green). Examples of TVN features observed are indicated as extended membrane cluster (mem. Cluster, green arrow), tubule (blue arrow), and vesicle (magenta arrow). Scale bar is 10 µm.

Supplementary Figure 3
*P. vivax* TVN features 8 dpi. **(A)** Representative confocal images depicting *P. vivax* parasites in PHH at 8 dpi with tubules associating with the host nucleus. Cells stained with DAPI (blue) and anti-*Pv*UIS4 (green). Scale bar is 10 µm.

Supplementary Figure 4
*P. vivax* TVN displays varying features throughout liver stage infection. EEFs displaying one or more TVN feature throughout *P. vivax* infection of PHH (2–10 dpi). Data reported as mean ± SEM (>30 parasites assessed, n=2 biological replicates).

Supplementary Figure 5 Antibody controls for AQP3 in *P. vivax* and *P. berghei* **(A)** Representative confocal images of *P. vivax* EEFs stained with *Pv*UIS4 (green) and donkey anti-rabbit Alexa Fluor^®^ 568-conjugated secondary antibody (red) without prior anti-AQP3 primary staining. **(B)** Representative widefield microscopy images of *P. berghei* liver stage infections in wild type and ΔAQP3 HuH7 cells. Cells stained with DAPI (blue), anti-*Pb*UIS4 (green), and anti-*Hs*AQP3 (red). Scale bar is 10 µm. **(C)** Representative confocal images of *P. berghei* EEFs stained with DAPI (blue), *Pb*UIS4 (green), and AQP3 primary & secondary antibody (red). **(D)** Representative confocal images of *P. berghei* EEFs stained with DAPI (blue), *Pb*UIS4 (green) and AQP3 primary antibody (red) without secondary antibody staining. **(E)** Representative confocal images of *P. berghei* EEFs stained with DAPI (blue), *Pb*UIS4 (green) and donkey anti-rabbit Alexa Fluor^®^ 568-conjugated secondary antibody (red) without prior anti-AQP3 primary staining. **(A–E)** Scale bar is 10 µm.

Supplementary Figure 6 AQP3 localizes to the TVN during *P. berghei* liver stage infection. Representative widefield microscopy images of *P. berghei* liver stage infections exhibiting TVN features at 2 dpi. Cells stained with DAPI (blue), anti-*Pb*UIS4 (green), and anti-*Hs*AQP3 (red). *P. berghei*-infected HuH7 cells show localization of AQP3 to TVN features (yellow arrows). Scale bar is 10 µm.
