# Supplementary material for: Characterization of the Tubovesicular Network in Plasmodium vivax Liver Stage Hypnozoites and Schizonts
Source: Front Cell Infect Microbiol. 2021 Jun 14;11:687019. doi: 10.3389/fcimb.2021.687019 (PMC8236947; doi:10.3389/fcimb.2021.687019)
Supplement: Supplementary Figure 1 — P. vivax variation in size 8 dpi. (A) Representative confocal images of P. vivax hypnozoites in PHH at 8 dpi, defined by EEF area < 60 µm2. (B) Representative confocal images of P. vivax schizonts in PHH at 8 dpi, defined by EEF area > 200 µm2. (A, B) Cells stained with DAPI (blue) and anti-PvUIS4 (green). Scale bar is 10 µm. [file DataSheet_1.pdf]

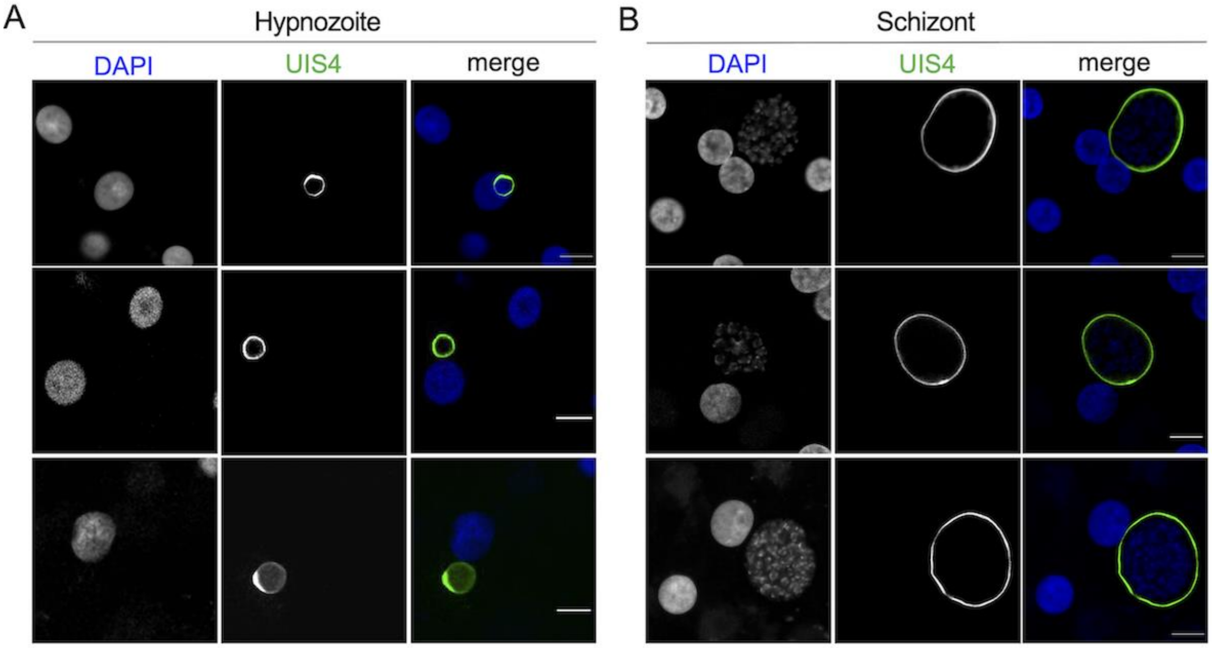

Supplemental Figure 1. *P. vivax* variation in size 8 dpi. (A) Representative confocal images of *P. vivax* hypnozoites in PHH at 8 dpi, defined by EEF area < 60 μm^2. (B) Representative confocal images of *P. vivax* schizonts in PHH at 8 dpi, defined by EEF area > 200 μm^2. (A-B) Cells stained with DAPI (blue) and anti-*Pv*UIS4 (green). Scale bar is 10 μm.

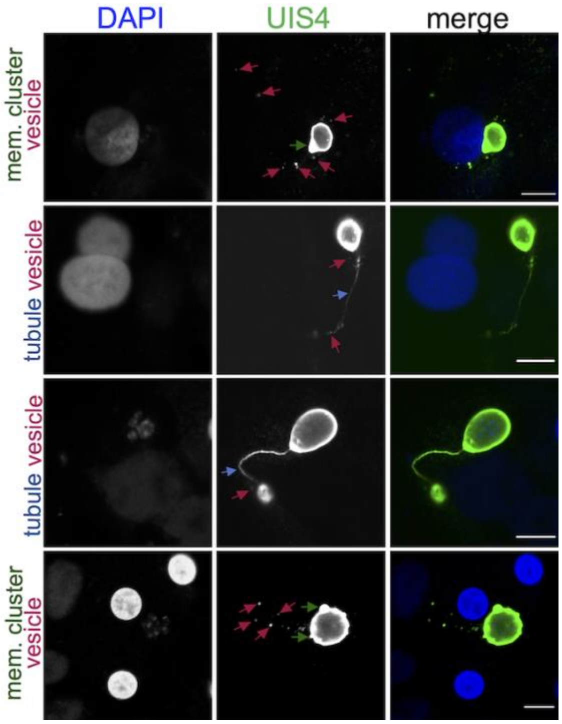

Supplemental Figure 2. *P. vivax* TVN+ EEFs contain multiple features. Confocal images of *P. vivax* EEFs with TVN features in PHH at 8 dpi. Cells stained with DAPI (blue) and anti-*Pv*UIS4 (green). Examples of TVN features observed are indicated as extended membrane cluster (mem. Cluster, green arrow), tubule (blue arrow), and vesicle (magenta arrow). Scale bar is 10 µm.

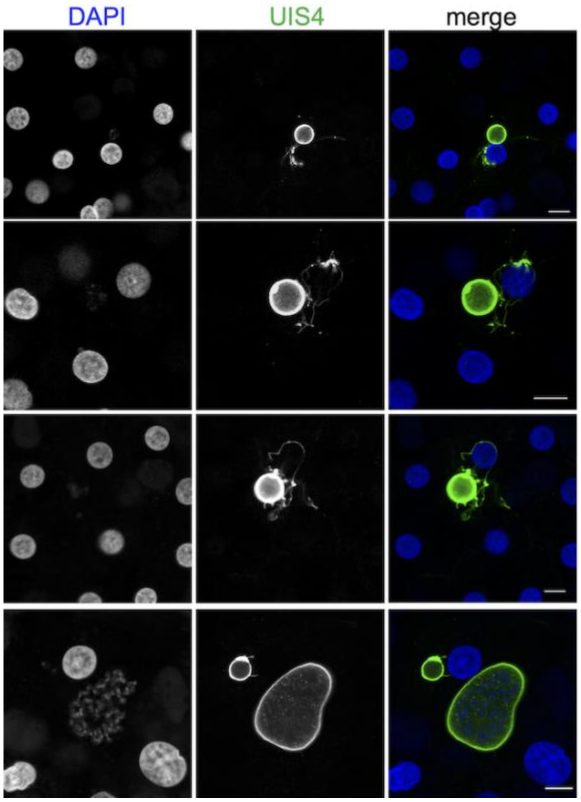

Supplemental Figure 3. *P. vivax* TVN features 8 dpi. (A) Representative confocal images depicting *P. vivax* parasites in PHH at 8 dpi with tubules associating with the host nucleus. Cells stained with DAPI (blue) and anti-*Pv*UIS4 (green). Scale bar is 10 µm.

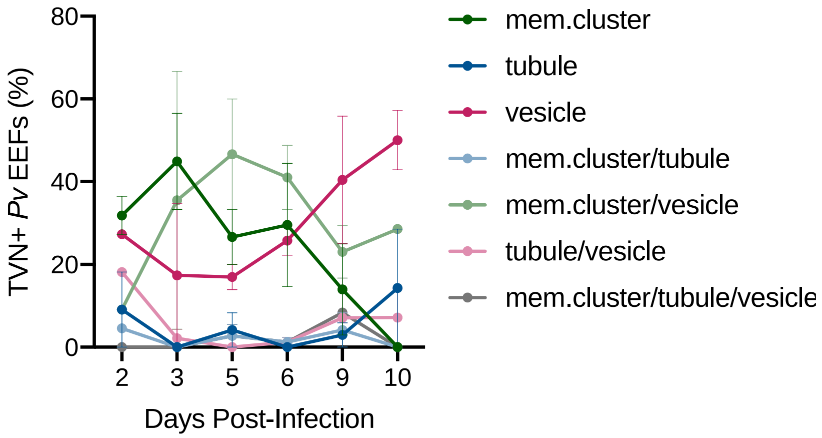

Supplemental Figure 4. *P. vivax* TVN displays varying features throughout liver stage infection. EEFs displaying one or more TVN feature throughout *P. vivax* infection of PHH (2–10 dpi). Data reported as mean ± SEM (>30 parasites assessed, n=2 biological replicates).

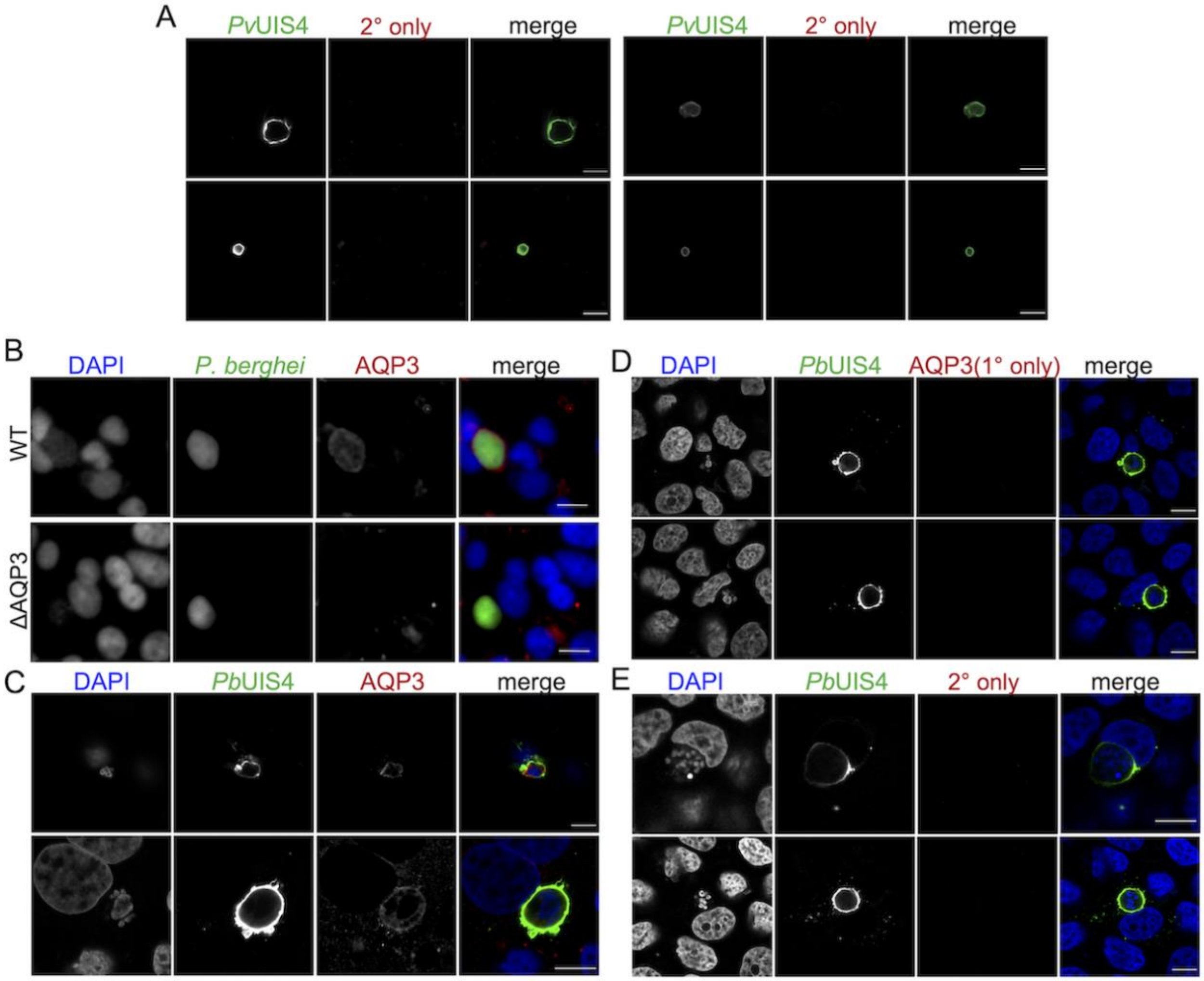

Supplemental Figure 5. Antibody controls for AQP3 in *P. vivax* and *P. berghei*. (A) Representative confocal images of *P. vivax* EEFs stained with *Pv*UIS4 (green) and donkey anti-rabbit Alexa Fluor® 568-conjugated secondary antibody (red) without prior anti-AQP3 primary staining. (B) Representative widefield microscopy images of *P. berghei* liver stage infections in wild type and ΔAQP3 HuH7 cells. Cells stained with DAPI (blue), anti-*Pb*UIS4 (green), and anti-*Hs*AQP3 (red). Scale bar is 10 µm. (C) Representative confocal images of *P. berghei* EEFs stained with DAPI (blue), *Pb*UIS4 (green), and AQP3 primary & secondary antibody (red). (D) Representative confocal images of *P. berghei* EEFs stained with DAPI (blue), *Pb*UIS4 (green) and AQP3 primary antibody (red) without secondary antibody staining. (E) Representative confocal images of *P. berghei* EEFs stained with DAPI (blue), *Pb*UIS4 (green) and donkey anti-rabbit Alexa Fluor® 568-conjugated secondary antibody (red) without prior anti-AQP3 primary staining. (A-E) Scale bar is 10 µm.

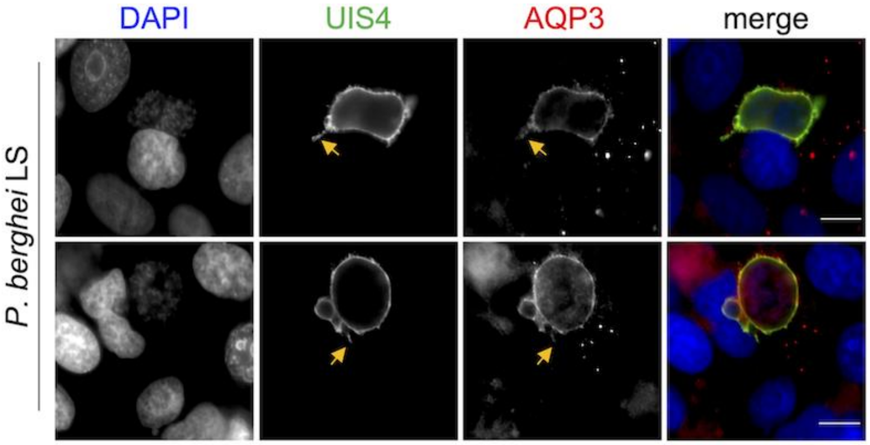

Supplemental Figure 6. AQP3 localizes to the TVN during *P. berghei* liver stage infection. Representative widefield microscopy images of *P. berghei* liver stage infections exhibiting TVN features at 2 dpi. Cells stained with DAPI (blue), anti-*Pb*UIS4 (green), and anti-*Hs*AQP3 (red). *P. berghei*-infected HuH7 cells show localization of AQP3 to TVN features (yellow arrows). Scale bar is 10 µm.
